# Patient-Centered Priorities for Older Adults in Ambulatory Care

**DOI:** 10.1001/jamanetworkopen.2025.35769

**Published:** 2025-10-06

**Authors:** Nicholas K. Schiltz, Bootan Ahmed, Heba M. Aldossary, Grace Q. Armstrong, Sherry A. Greenberg, Anna E. Bender, Anne Pohnert, Mary A. Dolansky

**Affiliations:** 1 Frances Payne Bolton School of Nursing, Case Western Reserve University, Cleveland, Ohio; 2 Department of Nursing, Prince Sultan Military College of Health Sciences, Dhahran, Saudi Arabia; 3 Hunter-Bellevue School of Nursing, Hunter College, City University of New York, New York, New York; 4 School of Medicine, Department of Pediatrics, University of Washington, Seattle; 5 MinuteClinic, CVS Pharmacy, CVS Health, Woonsocket, Rhode Island

## Abstract

This cross-sectional study investigates frequency of responses about health care priorities among older adults in ambulatory care.

## Introduction

Age-Friendly Health Systems (AFHS) is an initiative focused on integrating the 4Ms framework (What Matters, Medication, Mentation, and Mobility) in health care settings to enhance evidence-based care for older adults.^1^ Central to this framework is understanding patient preferences and goals by asking what matters most, fostering patient-centered care, and improving health outcomes.^2,3^ Despite the importance of older adults’ specific priorities in outpatient settings, these priorities remain underexplored. This study aimed to describe what matters most to older adults in walk-in ambulatory care clinics.

## Methods

This retrospective cross-sectional study used electronic health record data from a national network of walk-in ambulatory care clinics that implemented the 4Ms framework systemwide in May 2020 as part of routine care for all patients ages 65 and older.^4,5^ The study population included patients aged 65 and older who had in-person clinical encounters between January 2021 and March 2024 and were asked the open-ended question “What matters most to you?” to align 4Ms actions with their priorities. Clinicians documented responses into 1 of 5 predefined electronic health record categories established with stakeholder input during the preimplementation phase: social activities and inclusiveness, health, family togetherness, independence, and other. Further details on implementation planning,^5^ clinician training,^6^ documentation workflow, assessment rate, and 4Ms delivery^4^ are available in prior publications or eMethods 1 and 2 in Supplement 1. We used χ^2^ tests to assess differences in response categories (ie, count) for the evaluation period by age, self-reported race and ethnicity, sex, and year. Data were analyzed with R statistical software version 4.2.2 (R Project for Statistical Computing). All tests were 2-sided, with a significance level of α = .05. Reporting followed the STROBE reporting guideline. This study received institutional review board approval from Case Western Reserve University with a waiver of HIPAA authorization and informed consent under the 2018 Common Rule for research involving no more than minimal risk to participants.

## Results

Among 388 046 patients queried on what matters (2.7% Asian, 5.0% Black, 4.7% Hispanic, and 75.8% White; 64.5% female; 65.9% aged 65-74 years) (Table), the most common response was social activities and inclusiveness (48.6%), followed by health (21.0%), independence (17.0%), and family togetherness (10.5%). The distribution of responses was similar across subgroups. However, there were shifts in responses from 2021 to 2024 (Figure), such that social activities and inclusiveness increased from 38.6% to 54.9%, while health (26.3% to 19.1%) and family togetherness (16.8% to 6.3%) decreased (all *P* < .001). Responses for independence showed more modest fluctuations. The assessment rate increased from 21.8% in 2021 to 53.4% in 2024, while demographics of individuals assessed were similar to those not assessed (Table).

**Table.** Demographic Characteristics

Characteristic	Patients, No. (%)	Patients with response, % (95% CI)^a^						*P* value^b^
		Social activities and inclusiveness	Health	Independence	Family togetherness	Other	Refused	
Total	388 046 (100)	48.6 (48.4-48.8)	21.0 (20.9-21.1)	17.0 (16.9-17.1)	10.5 (10.4-10.6)	1.0 (1.0-1.0)	1.9 (1.9-1.9)	
Age group, y								
65-74	255 765 (65.9)	49.4 (49.2-49.6)	20.9 (20.7-21.1)	16.3 (16.2-16.4)	10.5 (10.4-10.6)	0.9 (0.9-0.9)	1.9 (1.8-2.0)	<.001
75-84	104 130 (26.8)	47.9 (47.6-48.2)	21.1 (20.9-21.3)	18.1 (17.9-18.3)	10.1 (9.9-10.3)	1.0 (0.9-1.1)	1.8 (1.7-1.9)	
≥85	28 151 (7.3)	44.2 (43.6-44.8)	21.3 (20.8-21.8)	19.1 (18.6-19.6)	11.6 (11.2-12.0)	1.3 (1.2-1.4)	2.5 (2.3-2.7)	
Sex								
Male	137 783 (35.5)	47.1 (46.8-47.4)	21.6 (21.4-21.8)	17.8 (17.6-18.0)	10.5 (10.3-10.7)	1.0 (0.9-1.1)	2.0 (1.9-2.1)	<.001
Female	250 145 (64.5)	49.5 (49.3-49.7)	20.7 (20.5-20.9)	16.6 (16.5-16.7)	10.5 (10.4-10.6)	0.9 (0.9-0.9)	1.8 (1.7-1.9)	
Unknown	118 (<0.1)	48.3 (39.3-57.3)	18.6 (11.6-25.6)	16.1 (9.5-22.7)	14.4 (8.1-20.7)	NA^c^	NA^c^	
Race and ethnicity^d^								
American Indian or Alaska Native	972 (0.3)	46.2 (43.1-49.3)	21.1 (18.5-23.7)	19.0 (16.5-21.5)	10.2 (8.3-12.1)	1.2 (0.5-1.9)	2.3 (1.4-3.2)	<.001
Asian	10 408 (2.7)	45.7 (44.7-46.7)	23.4 (22.6-24.2)	15.3 (14.6-16.0)	12.1 (11.5-12.7)	1.2 (1.0-1.4)	2.4 (2.1-2.7)	
Black or African American	19 489 (5.0)	49.4 (48.7-50.1)	20.8 (20.2-21.4)	17.1 (16.6-17.6)	10.2 (9.8-10.6)	1.0 (0.9-1.1)	1.5 (1.3-1.7)	
Hispanic	18 165 (4.7)	47.7 (47.0-48.4)	22.3 (21.7-22.9)	15.4 (14.9-15.9)	12.2 (11.7-12.7)	.8 (0.7-0.9)	1.6 (1.4-1.8)	
Native Hawaiian and Other Pacific Islander	510 (0.1)	48.8 (44.5-53.1)	19.6 (16.2-23.0)	16.7 (13.5-19.9)	12.0 (9.2-14.8)	NA^c^	NA^c^	
White	294 330 (75.8)	49.1 (48.9-49.3)	20.7 (20.6-20.8)	17.2 (17.1-17.3)	10.2 (10.1-10.3)	0.9 (0.9-0.9)	1.9 (1.9-1.9)	
Other	4397 (1.1)	47.2 (45.7-48.7)	21.9 (20.6-23.1)	16.6 (15.5-17.8)	11.2 (10.3-12.2)	1.3 (1.0-1.7)	1.8 (1.4-2.3)	
Unknown	30 679 (7.9)	45.2 (44.6-45.7)	22.2 (22.0-22.9)	17.1 (16.7-17.5)	11.9 (11.5-12.2)	1.4 (1.2-1.5)	2.1 (1.9-2.2)	
Patient refused	9096 (2.3)	48.2 (47.2-49.2)	21.6 (20.8-22.4)	16.5 (15.7-17.3)	10.5 (9.9-11.1)	0.9 (0.7-1.1)	2.3 (2.0-2.6)	

Abbreviation: NA, not applicable.

^a^ Frequency of responses to what matters among older adults visiting clinic locations between January 2021 and March 2024. There were 599 208 individuals not assessed (372 726 female [62.2%]; 1459 American Indian or Alaska Native [0.2%], 21 192 Asian [3.5%], 33 841 Black [5.6%], 30 447 Hispanic [5.1%], 1125 Native Hawaiian and Other Pacific Islander [0.2%], 432 936 White [72.3%], and 8024 other [1.3%]; 406 153 ages 65-74 years [67.8%] and 139 956 ages 75-84 years [25.0%]).

^b^ χ^2^ test.

^c^ Suppressed due to low counts.

^d^ Race and ethnicity categories were self-reported and derived from patient electronic health records. Patients who denoted their ethnicity as Hispanic were categorized as Hispanic regardless of racial identity. Patients reporting all other race and ethnicity categories listed did not identify as Hispanic. Data on race and ethnicity were included to ensure that findings reflected experiences of diverse patient groups.

**Figure.**
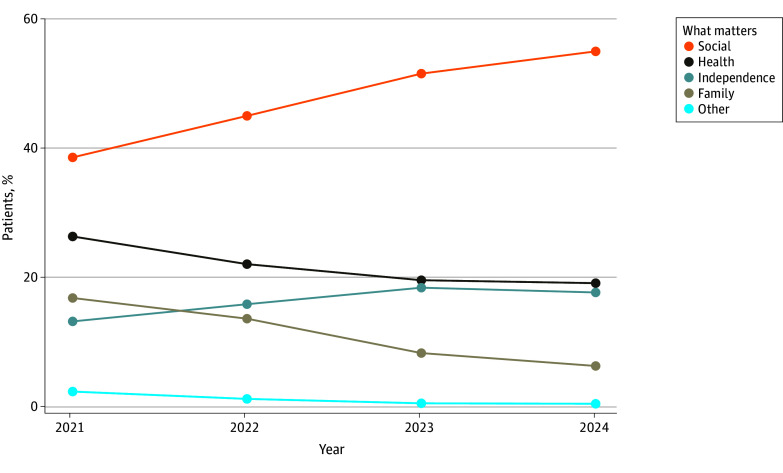
Trends in What Matters Most to Older Adults, 2021-2024 The distribution of what matters response frequency counts (%) among 388 046 older adults visiting clinic locations between January 2021 and March 2024 is shown. Statistically significant (*P* < .001) differences in responses by year were shown by χ^2^ tests. The number of assessments per year were as follows: 49 630 of 228 025 age-friendly eligible appointments (21.8%) in 2021, 99 627 of 306 174 eligible appointments (32.5%) in 2022, 187 362 of 356 712 eligible appointments (52.5%) in 2023, and 51 427 of 96 295 eligible appointments (53.4%) in the first quarter of 2024.

## Discussion

To our knowledge, this cross-sectional study is the largest report of what matters most to patients in ambulatory care settings. Among assessed patients, approximately 80% identified something other than their own health as what mattered most to them. Further research is needed to determine whether consistency across age, race and ethnicity, and sex subgroups is specific to patients in ambulatory care and generalizable to other settings.

Despite the large, geographically diverse study population representing hundreds of ambulatory walk-in clinics across 35 states, findings may not be generalizable to other health care settings. In addition, predefined categories may not fully capture what matters most; however, the low other selection rate (1.0%) suggests that predefined categories reflected most responses. While the simplicity of a single response in a broad category offers a starting point for understanding patient priorities, it may not fully capture the complexity of individual needs and may be insufficient to develop a comprehensive care plan.

This large study in clinical practice of older adult patients in ambulatory care across the US found that social activities and inclusiveness were often top patient priorities. Identifying what matters is essential for providing person-centered care, guiding clinical visits, tailoring care plans, and providing a starting point to foster further patient engagement. These insights highlight the need for health care systems to adopt strategies like the 4Ms framework to align care with patient priorities and values.

## Notes

Supplement 1. eMethods 1. Documentation Workflow eMethods 2. Assessment Rates

Supplement 2. Data Sharing Statement
