# Supplementary material for: Patient-Centered Priorities for Older Adults in Ambulatory Care
Source: JAMA Netw Open. 2025 Oct 6;8(10):e2535769. doi: 10.1001/jamanetworkopen.2025.35769 (PMC12501808; doi:10.1001/jamanetworkopen.2025.35769)
Supplement: Supplement 1. — eMethods 1. Documentation Workflow eMethods 2. Assessment Rates [file jamanetwopen-e2535769-s001.pdf]

Supplemental Online Content

Schiltz NK, Ahmed BH, Aldossary HM, et al. Patient-centered priorities for older adults in ambulatory care. *JAMA Netw Open*. 2025;8(10):e2535769. doi:10.1001/jamanetworkopen.2025.35769

eMethods 1. Documentation Workflow

eMethods2. Assessment Rates.

This supplemental material has been provided by the authors to give readers additional information about their work.

© 2025 Schiltz NK et al. *JAMA Network Open*.

eMethods 1. Documentation Workflow

Documentation Workflow for Providers Selecting 'What Matters' Most to Older Adult Patients

1. Provider asks patients "What Matters Most to you?"
2. If the answer is not clear, the provider is instructed to ask for examples.
3. Providers are given five pre-defined choices in the Electronic Health Record and an option for Refusal.
   a. Family Togetherness, Social activities/Inclusiveness, Health, Independence, Other, or Patient Declined.
   b. If a patient's response does not fit into one of four predefined categories, 'Other' may be selected, triggering a Free Text Response in the Electronic Health Record.
4. Providers are instructed to explain that asking 'What Matters Most' is aimed to align the treatment plan with their response and to promote active participation in their own health care.

eMethods2. Assessment Rates.

Between January 2021 and March 2024, the "What Matters" question was posed to 388,046 patients, representing 39.3% of the 987,206 age-friendly eligible visits. An age-friendly eligible visit is defined as any in-person clinic visit among adults aged 65 and older, excluding Express Lane visits, which are streamlined visits for vaccines, TB testing, and Vitamin B12 injections. A comparison of patients who were assessed versus those who were not revealed that the demographic characteristics of both groups are generally similar.

© 2025 Schiltz NK et al. *JAMA Network Open*.
