# Supplementary material for: Patient-Centered Priorities for Older Adults in Ambulatory Care
Source: JAMA Netw Open. 2025 Oct 6;8(10):e2535769. doi: 10.1001/jamanetworkopen.2025.35769 (PMC12501808; doi:10.1001/jamanetworkopen.2025.35769)
Supplement: Supplement 2. — Data Sharing Statement [file jamanetwopen-e2535769-s002.pdf]

Data Sharing Statement

Schiltz. Patient-Centered Priorities for Older Adults in Ambulatory Care. *JAMA Netw Open*. Published online Oct 6, 2025. doi:10.1001/jamanetworkopen.2025.35769

Data
Data available: No

Additional Information
Explanation for why data not available: Data were accessed under a data use agreement with CVS Health Corporation that prohibits data sharing.
